# Food and Inflammatory Bowel Diseases: A scoping review on the impact of food on patients’ psychosocial quality of life

**DOI:** 10.1111/hsc.13755

**Published:** 2022-02-17

**Authors:** Lorenzo Palamenghi, Polina Figliuc, Salvatore Leone, Guendalina Graffigna

**Affiliations:** ^1^ EngageMinds HUB – Consumer Food & Health Engagement Research Center Università Cattolica del Sacro Cuore Milan Italy; ^2^ Department of Psychology Università Cattolica del Sacro Cuore Milan Italy; ^3^ Faculty of Agriculture, Food and Environmental Sciences Università Cattolica del Sacro Cuore Cremona Italy; ^4^ AMICI Onlus Associazione nazionale per le Malattie Infiammatorie Croniche dell’Intestino Milano Italy

**Keywords:** diet, food consumption, food restrictions, IBD, psychosocial wellbeing, quality of life

## Abstract

Growing bodies of literature show that a controlled diet is important in controlling the symptoms of Inflammatory Bowel Diseases (IBD). This leads patients to avoid foods considered potentially harmful. However, food is not just a nutrient but entails a series of hedonistic, cultural and social values. Thus, there is the concern that having to renounce certain foods might exert an impact on patients’ psychosocial quality of life, particularly in younger patients. The aim of this paper is to review the existing literature to address which aspects of the patients’ quality of life are affected by food restrictions. A scoping review was carried out. Five different databases were searched in January 2021. Retrieved papers were then screened to only include the relevant studies. Data were extracted and the main results of the studies were charted. A thematic analysis was carried out on the main results to identify the areas of psychosocial quality of life more often impacted by the food restrictions. From the initially identified 1967 unique entries, 14 studies were included. Results show that the perceived importance of food in controlling symptoms is confirmed by patients’ accounts. The most common strategy adopted was, thus, the avoidance of trigger foods. The thematic analysis revealed three domains that are impacted by these restrictions: psychological quality of life, social life, family sphere. This study highlights the impact that food restrictions exert on IBD patients’ quality of life, and warrants further studies to fill existing gaps, in particular regarding younger patients.

**What is known about this topic?**

- Food is a known an important factor in determining IBD symptoms;
- Patients with Inflammatory Bowel Diseases have to avoid certain foods which they believe could worsen their symptoms;
- Little guidance is provided in helping patients understand which foods should be avoided.

**What this paper adds?**

- The psychosocial consequences of food restrictions have been investigated by a number of studies, both qualitative and quantitative;
- The necessity to control diet and avoid certain foods exerts an impact on three different aspects of patients’ lives: personal and psychological wellbeing, social life, and family sphere;
- Several gaps exist however regarding the psychosocial impact of food restriction on younger patients.

## 1 INTRODUCTION

Inflammatory Bowel Diseases (IBD) are a group of chronic conditions affecting the gastrointestinal system. These diseases include patients affected by Crohn's Disease (CD) and Ulcerative Colitis (UC), as well as a minority of patients with Indeterminate Colitis (IC). These diseases cause a variety of symptoms, even though the severity and frequency of symptoms vary across individuals and in time, and can exert a strong impact on patients’ quality of life. A recent review found that the highest reported prevalence and incidence of IBD were in European countries and in North America (Ng et al., 2017), even though since 1990 the incidence has been rising even in other continents, as industrialisation increased. Indeed, in 2008 the total economic burden of Crohn's Disease alone has been estimated at 10.9–15.5 billion dollars in the US, and between 2.1–16.7 billion euros in Europe (Yu et al., 2008). The relevance of these diseases for public health led to growing bodies of research focused on identifying the underlying causes that lead to a worsening in the frequency and severity of IBD patients’ symptoms: among the various factors that might impact patients’ symptoms, several studies highlighted the role of food and diet (Hou et al., 2014; Kinsey & Burden, 2016; Mehrabani et al., 2017). Indeed, the management of IBD symptoms through the implementation of dietary therapies aimed at modulating the intestinal microbiome is becoming a rather common method (Green et al., 2019). Patients themselves seem to frequently report an association between food and symptoms (Cohen et al., 2013). Nevertheless, even though the implementation of diets and food restrictions might help the patients to relieve their symptoms, this might actually come to a cost: from a psychological perspective, food is not only a nutrient but has several implications for people's identity and social life (Fischler, 1988; Lupton, 1994), though more marked in certain cultures (Rozin, 2005). Eating, indeed, is a fundamental part of our lives not only because it allows us to introduce nutrients and sustain our bodies, but also determines our own cultural identity (‘Tell me what you eat, and I will tell you who you are’) and is oftentimes a central activity in many important gatherings (Caplan, 1997). Thus, the dietary restrictions imposed by IBD might impact patients’ possibility to both enjoy food and the social dimension it entails. This might be particularly relevant and worrying for younger patients (children and adolescents), as the potentially impoverished social life might compromise their psychosocial development; additionally, the very fact that they cannot behave (in relationships to food) like their peers might pose a threat to their own self‐representation and self‐esteem.

Thus, it is important to assess the impact that dietary restrictions might exert on IBD patients’ quality of life and psychosocial wellbeing, particularly regarding younger patients, as this will allow to highlight potential sources of social and psychological distress and ultimately address the more critical areas to reduce patients’ burden and to sustain their engagement in self‐management.

The aim of the present paper is to map the scientific literature in order to map the existing evidence regarding the aspects of food that impact on the psychosocial wellbeing and the quality of life of patients with Inflammatory Bowel Disease.

## 2 MATERIALS AND METHODS

In order to answer our research question, we conducted a scoping review, as defined by Armstrong et al. (2011). We preferred a scoping review instead of a systematic review as this is a type of literature review that allows a broader exploration of a certain topic of interest, and a less focused research question. Since we were interested in exploring the ‘state of art’ of the knowledge regarding how food restrictions and diets in IBD impact on the psychosocial wellbeing and the quality of life of patients, we decided that a scoping review was preferable.

To carry out our study in a rigorous, though flexible, way, we referred to a framework describing a 4‐step process (Arksey & O’Malley, 2005):

1. identification of the possibly relevant studies by consulting a selection of the most relevant scientific citation databases;
2. selection of the potentially relevant studies by screening the titles and abstracts of the candidate articles identified in the databases;
3. data extraction and analysis from the eligible papers, and finally.
4. data charting and report.

### 2.1 Identification of the possibly relevant studies

To identify all the potentially relevant articles to be included in our review, we interrogated the most important scientific databases in the medical and psychological field, namely: Scopus, Web of Science Core Collection, Pubmed, EBSCO_ Cochrane Central Register of Controlled Trials, and PsycINFO. The search was conducted on January 7, 2021, including only articles written in English. The research string was composed of three main queries, each composed of different synonyms (connected with ‘OR’), and queries were connected to each other with ‘AND’ connectors. The three queries regarded, respectively: ‘Inflammatory Bowel Disease’, ‘Food’, and ‘psychosocial wellbeing’. All the identified studies were then imported into a reference manager (Mendeley) and checked for duplicates.

### 2.2 Screening and selection of relevant studies

The identified articles were then screened applying the following inclusion/exclusion criteria to the title and abstract of each publication; to be included, the article had to:

1) discuss the perception of the disease‐food relationship in IBD patients, its psychosocial outcomes, and the quality of life derived from such relationship (clinical trials and publications that evaluated specific dietary or medical treatments efficacy, and articles that considered merely the nutrients and chemical characteristics of food, were excluded);
2) be in English and available;
3) be an original article (non‐peer‐reviewed articles, opinions, letters and reviews were excluded).

Two researchers of the team (LP and PF) screened the identified articles by reading titles and abstracts. Conflicts regarding inclusion or exclusion were resolved by consensus.

### 2.3 Data extraction, collation and analysis

From the included studies we extracted three types of data:

1. Bibliometric data regarding the paper (reference, year of publication, journal and subject area of the journal);
2. Methodological data (quantitative or qualitative research, tools used, sample characteristics, nationality);
3. Main results of the studies.

In particular, regarding the results of the included study, we conducted a qualitative thematic analysis to identify the main themes that emerged from qualitative studies and/or were investigated in the quantitative studies. Thus, results from the included studies are reported inside the specific categories that were identified in our thematic analysis.

## 3 RESULTS

### 3.1 Identification and selection of relevant studies

**FIGURE 1:**
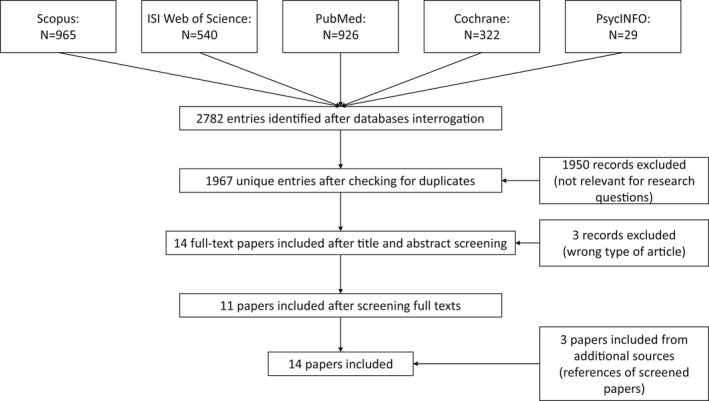
PRISMA flow chart

Figure 1 shows the results of the screening procedure. 2782 records were identified through the database search and exported into the citation manager (Mendeley). After removing duplicates (via automatic tools and manual check), 1967 publications remained.

After the first screening (title & abstract), 14 articles were included as potentially relevant and screened in full text. 3 of these papers were then excluded as they did not match the inclusion criteria. However, 3 additional papers—which were not found by our research string but retrieved by the inspection of included papers’ reference lists—were later included, as relevant. The total number of papers included for data extraction was then 14.

### 3.2 Characteristics of the included studies

The bibliometric data and principal characteristics of the 14 articles included in the review are summarised in Table 1. The oldest included article that investigates the impact of food in IBD patients is that from Jowett and colleagues from 2004 (Jowett et al., 2004), while the most recent is from 2021 (Crooks et al., 2021). All the included studies have been published in journals focused on the health sciences area: in particular, 3 articles were published in journals focused on gastroenterology (Crooks et al., 2021; Palant et al., 2015; De Vries et al., 2019), and an additional two in journals specifically dedicated to IBD (Limdi et al., 2016; Zallot et al., 2013). The remaining articles are from journals dedicated to medicine in general, nursing, or quality of life.

**TABLE 1:** Bibliometric characteristics of the included papers

Autor(s)	Title	Year	Journal	Journal subject area^a^
Alexakis, C.; Nash, A.; Lloyd, M.; Brooks, F.; Lindsay, J. O.; Poullis, A.	Inflammatory bowel disease in young patients: challenges faced by black and minority ethnic communities in the UK	2015	Health & Social Care in the Community	Medicine: Health Policy; Public Health, Environmental and Occupational Health. Social Sciences: Social Sciences (miscellaneous); Social Work; Sociology and Political Science.
Chuong, K. H.; Haw, J.; Stintzi, A.; Mack, D. R.; O'Doherty, K. C.	Dietary strategies and food practices of paediatric patients, and their parents, living with inflammatory bowel disease: a qualitative interview study	2019	International journal of qualitative studies on health and wellbeing	Medicine: Health Policy. Nursing: Fundamentals and Skills; Gerontology; Issues, Ethics and Legal Aspects.
Crooks, B.; McLaughlin, J.; Matsuoka, K.; Kobayashi, T.; Yamazaki, H.; Limdi, J. K.	The dietary practices and beliefs of people living with inactive ulcerative colitis	2021	European Journal of Gastroenterology & Hepatology	Medicine: Gastroenterology; Hepatology.
Czuber‐Dochan, W.; Morgan, M.; Hughes, L. D.; Lomer, M. C. E.; Lindsay, J. O.; Whelan K.,	Perceptions and psychosocial impact of food, nutrition, eating and drinking in people with inflammatory bowel disease: a qualitative investigation of food‐related quality of life	2020	Journal of Human Nutrition and Dietetics	Medicine: Medicine (miscellaneous). Nursing: Nutrition and Dietetics.
De Vries J. H.M.; Dijkhuizen, M.; Tap, P.; Witteman, B. J.M.	Patient's Dietary Beliefs and Behaviours in Inflammatory Bowel Disease	2019	Digestive Diseases	Medicine: Gastroenterology; Medicine (miscellaneous).
Fletcher, P. C.; Schneider, M. A.	Is There Any Food I Can Eat? Living With Inflammatory Bowel Disease and/or Irritable Bowel Syndrome	2006	Clinical Nurse Specialist	Nursing: Advanced and Specialised Nursing; Assessment and Diagnosis; Leadership and Management; LPN and LVN
Guadagnoli, L.; Mutlu, E. A.; Doerfler, B.; Ibrahim A.; Brenner, D.; Taft, T. H.	Food‐related quality of life in patients with inflammatory bowel disease and irritable bowel syndrome	2019	Quality of Life Research	Medicine: Public Health, Environmental and Occupational Health
Jowett, S. L.; Seal, C. J.; Phillips, E.; Gregory, W.; Barton, J. R.; Welfare, M. R.	Dietary beliefs of people with ulcerative colitis and their effect on relapse and nutrient intake	2004	Clinical Nutrition	Medicine: Endocrinology, Diabetes and Metabolism. Nursing: Nutrition and Dietetics.
Limdi, J. K.; Aggarwal, D.; McLaughlin, J. T.	Dietary Practices and Beliefs in Patients with Inflammatory Bowel Disease	2016	Inflammatory Bowel Diseases	Medicine: Gastroenterology; Immunology and Allergy.
Marsh A.; Kinneally J.; Robertson T.; Lord A.; Young A.; Radford–Smith G.	Food avoidance in outpatients with Inflammatory Bowel Disease – Who, what and why	2019	Clinical Nutrition ESPEN	Medicine: Endocrinology; Diabetes and Metabolism. Nursing: Nutrition and Dietetics.
Palant A.; Koschack J.; Rassmann S.; Lucius‐Hoene G.; Karaus M.; Himmel W.	‘And then you start to loose it because you think about Nutella’: The significance of food for people with inflammatory bowel disease ‐ a qualitative study	2015	BMC Gastroenterology	Medicine: Gastroenterology; Medicine (miscellaneous).
Pituch‐Zdanowska A.; Kowalska‐Duplaga K.; Jarocka‐Cyrta E.; Stawicka A.; Dziekiewicz M.; Banaszkiewicz A.	Dietary Beliefs and Behaviours Among Parents of Children with Inflammatory Bowel Disease	2019	Journal of Medicinal Food	Medicine: Medicine (miscellaneous). Nursing: Nutrition and Dietetics.
Schneider Margaret A.; Jamieson A.; Fletcher P. C.	‘One sip won't do any harm…’: Temptation among women with inflammatory bowel disease/ irritable bowel syndrome to engage in negative dietary behaviours, despite the consequences to their health	2009	International Journal of Nursing Practice	Nursing: Nursing (miscellaneous).
Zallot C.; Quilliot D.; Chevaux J. B.; Peyrin‐Biroulet C.; Guéant‐Rodriguez R. M.; Freling E.; Collet‐Fenetrier B.; Williet *N*.; Ziegler O.; Bigard M. A.; Guéant J. L.; Peyrin‐Biroulet L.	Dietary Beliefs and Behaviour Among Inflammatory Bowel Disease Patients	2013	Inflammatory Bowel Diseases	Medicine: Gastroenterology; Immunology and Allergy.

^a^ Journal subject area was retrieved from: Scimago (https://www.scimagojr.com/ )

Regarding methodology, 6 articles describe qualitative research (Alexakis et al., 2015; Chuong et al., 2019; Czuber‐Dochan et al., 2020; Fletcher & Schneider, 2006; Palant et al., 2015; Schneider et al., 2009), 7 provide quantitative data (Crooks et al., 2021; Guadagnoli et al., 2019; Limdi et al., 2016; Marsh et al., 2019; Pituch‐Zdanowska et al., 2019; De Vries et al., 2019; Zallot et al., 2013) and the remaining one used a mixed methodology (Jowett et al., 2004). Three articles that were included in the review featured a sample with both IBD and IBS (Inflammatory Bowel Syndrome) patients (Fletcher & Schneider, 2006; Guadagnoli et al., 2019; Schneider et al., 2009). We decided to include these studies too, as the authors themselves considered IBD and IBS patients similar for what concerns the food and disease and the way food restrictions impact on their psychosocial wellbeing. Regardless, whenever it was possible, only results pertaining IBD patients were extracted and summarised from these studies. Only a minority of the included studies regarded young patients: in particular, 2 articles included adolescents (over 16 years old) along with adults (Alexakis et al., 2015; Czuber‐Dochan et al., 2020); in one study participants were children between 9 and 17 years old (along with their parents) (Chuong et al., 2019); finally, one study used the parents of young IBD patients as a proxy (Pituch‐Zdanowska et al., 2019). All the remaining 10 studies concerned only adult patients. All the studies were conducted in western countries; one article focused on black and minority ethnic communities in the UK (Alexakis et al., 2015). Table 2 describes the included studies’ methods and methodologies.

**TABLE 2:** included studies’ methodology, methods and sample

Reference	Methodology	Research methods	Sample size	Sample IBD type	Sample Age/Age groups	Sample Country
Alexakis et al., 2015	Qualitative study	Semi‐structured interviews with young people with IBD from black and minority ethnic groups	20	CD (13), UC (6), other (1)	16–24	UK
Chuong et al., 2019	Qualitative study	Semi‐structured interviews with children and their parents or grandparents	28	CD (23), UC (5)	Children and adolescents (9–17)	Canada
Crooks et al., 2021	Quantitative study	Questionnaire developed by the authors (27 questions)	208	UC	≥18	UK
Czuber‐Dochan et al., 2020	Qualitative study	Semi‐structured interviews conducted with people with IBD	28	CD (16), UC (12)	≥ 16	UK
De Vries et al., 2019	Quantitative study	Questionnaire developed by the authors (37 close‐ended questions)	294	CD (146), UC (148)	18–79	Netherlands
Fletcher & Schneider, 2006	Qualitative study	Semi‐structured interviews with women with IBD or IBS	8	2 UC, 1 UC+IBS, 5 IBS	18–22	Canada
Guadagnoli et al., 2019	Quantitative study	Survey including measures of Food‐Related Quality of Life, Health‐Related Quality of Life, disease activity, anxiety and depression	175	IBD (95), IBS (80)	18–70	USA
Jowett et al., 2004	Mixed method: qualitative assessment of nutritional beliefs and a quantitative assessment of nutritional intake	Interview at recruitment (beliefs) + food frequency questionnaire (nutritional intake) at recruitment and once a week for 1 year +validated disease activity index at recruitment and once a week for 1 year	191 (follow‐up complete in 183 patients)	UC	18–70	UK
Limdi et al., 2016	Quantitative study	Questionnaire developed by the authors (demographics +18 questions relating to dietary beliefs and food‐related behaviour)	400	CD (156), UC (205), not sure or no response (53)	>18	UK
Marsh et al., 2019	Quantitative study	Structured interviews with patients with IBD including nutritional assessment and evaluation of medical records	117	CD (50), UC (61), unspecified (6)	>18	Australia
Palant et al., 2015	Qualitative study (grounded theory)	Open‐end narrative interviews conducted with people with different IBD types, disease activities, and prior surgeries	42	CD (25), UC (15), IC (2)	Young adults, Middle aged and Aged people	Germany
Pituch‐Zdanowska et al., 2019	Quantitative study	Questionnaire developed by the authors administered to parents of children with IBD (demographics and disease characteristics +13 questions about dietary beliefs and practices +list of products avoided or that should be avoided)	155	CD (104), UC (51)	4–8	Poland
Schneider et al., 2009	Qualitative study (phenomenological study guided by heuristic inquiry)	Semi‐structured interviews with women with IBD or IBS +background questionnaire +food diary	3 IBD and 5 IBS	3 IBD (1 CD, 1 UC, 1 CD+UC), 5 IBS	18–23	Canada
Zallot et al., 2013	Quantitative study	Questionnaire developed by the authors (14 questions relating to dietary beliefs and dietary behaviour)	244	CD (177), UC (67)	>17	France

### 3.3 Main results

The studies included in our literature review confirm that patients perceive the diet as an important element to control the disease. Some studies reported that a large part of participants (31%–48%) believe that diet is a potential cause of their IBD (Crooks et al., 2021; Limdi et al., 2016), even though other studies reported lower percentages of patients who shared this point of view (13%–16%) (De Vries et al., 2019; Zallot et al., 2013). Nevertheless, the number of participants who believe that food habits could trigger a relapse is higher (between 33% and 58%) (De Vries et al., 2019; Zallot et al., 2013). In particular, the belief that certain foods can be the cause of relapses seems to be more prevalent in parents of children with IBD when compared with adults (Pituch‐Zdanowska et al., 2019). The authors also found that dietary beliefs seem to be perceived differently by parents depending on age and illness duration: parents of children with a longer history of IBD tended to respond more frequently that food could be a cause of the illness, and parents of older children expressed greater concern that dietary habits could trigger an IBD flare (Pituch‐Zdanowska et al., 2019).

Hence, many patients considered adapting their dietary intake to control the symptoms, prevent, or end the relapses faster; a large number of patients (21%–59%) reported diet as equally or more important than medicines in the management of the disease (Limdi et al., 2016; Marsh et al., 2019; De Vries et al., 2019). Furthermore, one study revealed that almost one patient in three is convinced that consuming nutritional supplements or specific food or drinks could prevent a relapse (Crooks et al., 2021). In accordance with these results, one qualitative study reported that many patients perceived a bi‐directional relationship between IBD and food: on the one hand, the disease could affect their dietary habits, on the other hand, dietary strategies could be considered an efficient way to manage the symptoms. Although in this study food was not reported as the direct reason for IBD flares, most patients believed that certain foods or food categories could worsen their symptoms, especially during active disease (Czuber‐Dochan et al., 2020).

The strategies more frequently reported are the avoidance of foods believed to be ‘triggers’, the assumption of ‘beneficial’ alimentary products, and the use of specific dietary treatments. Most of the included studies reported that IBD patients tend to avoid or reduce the intake of certain foods to reduce symptoms or prevent a relapse (Crooks et al., 2021; Limdi et al., 2016; Marsh et al., 2019; Pituch‐Zdanowska et al., 2019; De Vries et al., 2019; Zallot et al., 2013), even though it is not always easy to completely avoid these foods, which sometimes results in ‘cheating’, particularly in children (Chuong et al., 2019; Fletcher & Schneider, 2006; Palant et al., 2015; Schneider et al., 2009). The strategy of food avoidance is adopted by 59%–90% of participants and many of them avoid more than one dietary product, especially during relapses. Patients with CD tend to avoid significantly more foods than patients with UC both during active phases and remissions (Marsh et al., 2019). The results of almost all studies agree that the most omitted food categories are very common food categories, such as spicy foods (44%–81%), fat or strongly seasoned foods (32%–70%), food containing lactose, carbonated drinks, milk and other dairy products, raw vegetables, raw fruits, and fibres (Jowett et al., 2004; Marsh et al., 2019; De Vries et al., 2019; Zallot et al., 2013).

De Vries et al. (2019) also reported a list of foods, consumed by the participants, which were believed to be beneficial: wholemeal bread, tea, leafy vegetables, fatty fish, and poultry. Also, the use of dietary supplements, as well as the adoption of specific food exclusion diets, is reported by most studies as common in patients with IBD, especially during relapses (Crooks et al., 2021; Guadagnoli et al., 2019; De Vries et al., 2019). The main reasons for the use of supplements were to improve health and reduce fatigue (De Vries et al., 2019). Marsh and colleagues, conversely, reported that the majority of their participants did not follow any specific dietary pattern (Marsh et al., 2019). Crooks and colleagues reported that almost a quarter of the patients with UC that took part in their study had tried a specific whole food exclusion diet and 12% had tried more than one; the most followed were gluten‐free diet and lactose‐free diet (Crooks et al., 2021). Zallot et al. found that only 25% of their sample maintained a normal diet during relapse; more than half of the participants followed a low‐residue diet (Zallot et al., 2013). Other strategies to prevent symptoms emerged from the interviews were changing food preparation making dishes easier to digest, replacing ‘bad foods’ with ‘good foods’, eating slowly and moderating the intake of certain products without complete exclusion (Czuber‐Dochan et al., 2020; Fletcher & Schneider, 2006).

Regardless of this, there are some accounts of patients stating that they base their own food choices based solely on their preferences, regardless of the (foreseeable) consequence of having to go to the toilet more often, as they did not feel like they could deny themselves the pleasure of eating certain foods and they felt that eating ‘normal’ food and living as they did before the diagnosis made them feel more ‘normal’ (Czuber‐Dochan et al., 2020; Palant et al., 2015; Schneider et al., 2009).

Our thematic analysis revealed that these implemented strategies, the perceived necessity to renounce certain food categories and the attention that patients need to pay to food preparation and intake impact on three different aspects of their quality of life: personal and psychological wellbeing, social life, and family sphere.

Table 3 describes the quantitative studies’ main findings and qualitative studies themes, categorised into the 3 identified thematic categories.

**TABLE 3:** themes investigated by the authors

Reference	Dietary beliefs	Dietary strategies	Dietary behaviour/everyday practice	Dietary knowledge/support	Psychological factors
Alexakis et al., 2015			Patients reported difficulties with food types associated with their cultures, (e.g., not fasting during Ramadan); different meals to those of their family members; avoidance of large social functions.	Lack of awareness of IBD in primary care; general satisfaction of the IBD multidisciplinary team and the level of hospital service; language barriers affect parents’ capacity to provide appropriate support for their children	Bullying at school (disease‐specific harassment); feeling of anxiety and social exclusion caused by the avoidance of foods relevant to cultural identity; sense of guilt of patients towards their families for having to make a special effort to accommodate their dietary requirements
Chuong et al., 2019		food avoidance and moderation; following a specific diet; healthy eating	impact on grocery, shopping, meal planning, and cooking		maintaining routine and normality
Crooks et al., 2021	31% of participants believe that diet was the initiating factor of their UC and 37% are convinced that diet triggered a relapse of their disease. The main source of these beliefs is participants' own experience. The most commonly identified trigger foods are spicy foods (44%) and fatty foods (40%). Just 54% of participants believe that dietary advice during relapse and that during remission were different. 29% are convinced that consuming nutritional supplements or specific foods and drinks could prevent a relapse.	Most of the participants (59%) reported avoiding certain foods or drinks at least sometimes; 98% of them avoid more than one dietary product. Almost a quarter of participants (24%) had tried a specific whole food exclusion diet and 12% had tried more than one of this kind of diets.	28% of those who live with family (*n* = 164) avoided eating the same meal as the rest of the family at least sometimes. 21% of participants avoided eating out at list sometimes.	The main source that guides food avoidance is participants' own experience (90%), followed by healthcare professional (19%) and the internet (11%). For those who consumed specific foods, drinks or nutritional supplements (*n* = 34), their main source of information was their own experience (76%), internet (53%) and advice from healthcare professional (24%). Less than half of the participants (48%) reported being able to find dietary advice for UC and 60% of those that could, used internet as source of information.	
Czuber‐Dochan et al., 2020	Perception of the relationship between food and IBD: the disease affects the diet, but diet itself is perceived as a functional way to control the disease.	‘experimenting’ with food intake to manage symptoms; food avoidance, food exclusion and food substitution; replacing ‘bad foods’ with ‘good foods’; frequency of eating, portion sizes and planning ahead; healthy eating, vitamins and minerals; eating preferred food and dealing with consequences	Being organised, shopping, recipes and food preparation; impact on family, personal and professional life; social occasions and eating out	Not knowing enough; conflicting information regarding food in IBD; health professionals, family and friends as source of information and support; limited sources of information and support	Accepting new situation and ‘normalisation’; Being in control; missing the pleasure of being unrestricted about eating and drinking
De Vries et al., 2019	Only 13% of the patients think that diet is the most important cause of their IBD and 33% believe that nutrition plays an important role in causing relapse. However, 40% of all participants believe that adapting their dietary intake can end the relapse faster. 29% expect to gain more control over the disease through nutrition in the future. The majority of respondents (62%) reported that they successfully control their symptoms by adapting their nutrition, 30% of whom only during remission and 27% almost always. 59% of the patients believe diet to be either more (12%) or equally important (47%) compared to their medicine. 61% believed IBD decreases appetite, mainly only during relapse.	48% of participants reported to have followed a special diet for their IBD and 76% avoided certain foods in order to reduce symptoms. Most omitted food categories were spicy foods (75%), strongly seasoned foods (70%), carbonated drinks (56%), milk and other dairy products (52%). More than half of respondents (57%) consumed certain dietary products more frequently in order to have a beneficial effect on their disease symptoms. The most common food that was consumed more by 56% of the participants was wholemeal bread, followed by tea (47%), leafy vegetables (44%), fatty fish (42%) and poultry (39%). Dietary supplements were used by 68% of the patients.	Nutrition and lifestyle adaptations to reduce disease symptoms mainly reported by the participants included regular mealtimes (65.2%), sports and exercise (60.8%), more frequent smaller portions (42%) and relaxation (41%).	The majority of participants reported that the main source of their nutrition knowledge related to their IBD was based on own experience (81%), followed by the internet (37%), the dietician (25%) and the treating medical specialist in the hospital (24%). Of the participants who had received dietary advice, more than 70% were satisfied.	
Fletcher & Schneider, 2006		Avoidance of ‘trigger’ foods/beverages; use of pills to prevent an episode; pay the consequences after consuming ‘trigger’ foods; eating healthy; keep a food diary; continuous learning process (trial & error)	Being away from home and problems associated with food and travel		Uncertainty causes frustration; stressful situations trigger symptoms after eating
Guadagnoli et al., 2019	IBD patients reported better FRQoL than IBS patients, with a medium effect size (d = 0.56). IBD patients in remission demonstrated higher FRQoL than IBD and IBS patients with active disease. IBD and IBS patients with active disease did not differ in FRQoL.	Overall, IBS patients, compared with IBD patients, were more likely to use dietary treatments. Interestingly, self‐directed dietary therapy was most used by patients in this study, rather than adherence to a well‐described diet. Concurrent multiple diet use occurred in 33% of IBD and 36% of IBS patients at time of study; the maximum simultaneous diets used were three in 11% of subjects. The more diets a patient used, the poorer FRQoL for both, IBS and IBD patients. Less than half of subjects reported ever meeting with a dietitian regarding dietary treatment for their disease. IBD patients were more likely to have met with a dietitian. However, meeting with a dietitian did not translate to improved FRQoL.			
Jowett et al., 2004	39% of patients, when interviewed, believed that certain foods had been responsible for triggering a relapse at some time. Patients’ food beliefs were determined when they were in remission and related to their habitual diet. The majority (68%) of patients believed that food plays a role in their colitis and reported that they ate more or less of a particular food because of it. 49% avoided certain foods, 22% ate more of foods that they believed helped their colitis and 39% thought that certain foods triggered a relapse.	Of those who avoided certain foods (49% of participants), only 24% limited the intake of just one dietary product, the rest avoided two or more foods. The most common foods that patients avoided were milk or dairy products, then fruit and vegetables.		patients who believed that food was important had received dietary advice; most common source of advice was from dieticians.	
Limdi et al., 2016	48% believed that diet initiates the disease and 57% believed that food has a role in triggering a relapse. Dietary habits are perceived to be more important than medicines in the control of the disease for 28% of participants.	56% of participants modified their diet after the diagnosis and 68% avoided certain food types to prevent a relapse. The most avoided dietary products are spicy foods (45%) and fatty foods (32%). 60% of participants stated that these food categories are implicated in worsening of symptoms.	23% of participants do not share the same menu as the other members of the family and 20% refuse outdoor eating in order to prevent relapse.	Half participants (50%) stated that they had never received any nutritional advice; those who received any identified as the main sources of information dietician (31%) and gastroenterologist (17%). However, most of the participants (67%) are keen on receiving further dietary advice and the preferred sources of information are dietician (45%), IBD nurse specialist (36%), gastroenterologist (29%) and information leaflets (27%).	66% of participants stated that they deprived themselves of their favourite foods in order to prevent relapse and 73% reported that IBD changed their appetite and pleasure in eating. Appetite decreased during relapse and improved outside relapse. More CD patients felt that the disease affected their appetite (87% in CD versus. 66% in UC).
Marsh et al., 2019	, dietary habits versus. medicine	food avoidance (number and type of food groups avoided, reasons for food avoidance)		Source and confidence in dietary advice	Belief that IBD affected the appetite and pleasure of eating
Palant et al., 2015		food avoidance, ‘listen for their own bodies’, learning process: find out the food products they could tolerate, fear/aversion towards eating and drinking, risk of malnutrition	Difficulties when attending ceremonies; have to cook different meals for themselves and their families; concerns about travelling due to the lack of opportunities for cooking own food	professional help as further source of uncertainty (some patients found helpful the brochures given by their doctors, other did not feel supported by the health system)	Eating: between craving and aversion (difficult to abstain from certain products, eating preferred food and dealing with consequences, fear of eating/drinking); Being different (difficulties when attending ceremonial occasions/social events, eating differently from their family, concerns about travelling)
Pituch‐Zdanowska et al., 2019	Parents of children with a longer history of the illness indicated more frequently that food habits could cause their child's illness and parents of older children expressed greater fear that food can trigger an IBD flare.	Among foods that children with IBD avoided were fast food (83%), soft cheeses (83%), vegetable vinegar pickles (83%), hot spices and spicy foods (82%), and carbonated and noncarbonated soft drinks (79%).	Parents of the children with a longer history of the illness more frequently admitted that their child shared the same menu as the other members of the family. In the opinion of 44% parents, the disease was the reason why the child feared or refused outdoor dining, with girls avoiding outdoor dining more often than boys.	More than half of parents thought that children with IBD required care from a dietician and claimed that nutritional advice from a registered dietician was easily available. Almost all responders received nutritional instruction. As a source of knowledge about diet, parents most often mentioned the doctor (74.3%) and/or dietician (70.1%), but they also sought information from nonprofessional sources (84.7%).	Parents of children who suffered from IBD for a shorter period of time more often believed that children currently derive less pleasure from eating than before the illness. 1/3 of all participants believed that their children avoided some products they like because of fear of exacerbating the disease. 65% of respondents agreed that their child avoided foods they really liked: mainly fried dishes, sweets, fast food, milk and any milk products, and salty snacks.
Schneider et al., 2009					Giving into temptations (Cost–benefit analysis: denial/magical thinking; pursuit of normalcy, blatant disregard, purposeful actions; Physical and psychological reliance on medications: proactive behaviours (or over‐the‐counter medications as insurance, dependent behaviours (or over‐the‐counter medications and mind games); Awareness and timing of surroundings: comfort of home, fear of unfamiliar/uncontrollable surroundings)
Zallot et al., 2013	Just 16% of participants believed that diet could initiate the IBD, but most patients (58%) believed that food can be a risk factor in causing relapse.	Mostly avoided dietary products are too spicy foods (81%), too fatty foods (49%), raw vegetables (48%), carbonated beverages (45%), raw fruits (44%) and fibres (41%). During relapse, patients tended to exclude more foods and only 25% of participants maintained a normal diet; most respondents (52%) followed a low‐residue diet.	22% of respondents declared refusing outdoor dining for fear of causing relapse and 19% reported not sharing the same menu as other members of the family.	73% of participants reported having received nutritional advice. The main sources of dietary recommendations were dietitian (47%) and gastroenterologist (44%). More than half of participants (53%) would like to receive some advice on diet.	The majority of participants (48%) stated that the disease had changed their pleasure of eating. This is more evident in CD than in UC patients (54% versus. 38% respectively). Decreased appetite was reported during relapse compared to remission. 67% of participants reported avoiding certain foods they like in order to prevent relapse.

#### 3.3.1 Personal and psychological wellbeing

Even though avoiding trigger food seems to be a functional strategy to reduce the frequency of relapses and flares, in many studies emerged that patients also reported that having to pay a higher level of attention on food intake affects their own personal lives, as many of them reported having troubles in managing everyday activities, due to the need to adapt their diet to control IBD symptoms (Alexakis et al., 2015; Chuong et al., 2019; Czuber‐Dochan et al., 2020; Palant et al., 2015); indeed, many patients reported having to carefully plan their daily activities (e.g. food shopping, food preparation) in advance. This careful planning is often time consuming (Czuber‐Dochan et al., 2020), and the need to constantly pay attention and to restrain their food routine may actually lead to feelings of frustration, annoyance and, finally, distress (Czuber‐Dochan et al., 2020). Indeed, a study showed that the number of different diets a participant was following negatively correlated with his/her food‐related quality of life: this is worrying, as the same study observed that two IBD patients out of three were following at least a diet at the time of the study, and many of them were following multiple diets (Guadagnoli et al., 2019). In some cases, food restriction revealed a maladaptive strategy, leading to a continuous sense of fatigue and extreme weight loss (Czuber‐Dochan et al., 2020).

Another aspect that seems to impact on patients’ personal and psychological wellbeing is the uncertainty caused by the difficulty in finding trustable and reliable sources of information regarding food. Indeed, it seems that one of the most common ways to identify trigger foods is by experimenting, using a ‘trial and error’ process, and listening to one's own body (in both adult and paediatric patients) (Chuong et al., 2019; Czuber‐Dochan et al., 2020). However, this uncertainty and the fact that restrictions and sacrifices do not always work, make some patients feel frustrated, irritated and doubtful, potentially causing non‐adherence and a worsening in the symptoms due to a lack of control of the diet (Czuber‐Dochan et al., 2020; Palant et al., 2015). In particular, the participants in the study from Chuong and colleagues stated that the individuation of trigger foods is generally perceived as a personal experience and the list of these foods or food categories varied widely between young participants (Chuong et al., 2019). Indeed, personal experience seems to be one of the most common sources of information that patients need to rely on regarding nutrition and diet (Limdi et al., 2016; De Vries et al., 2019); in particular, in the study from Crooks and colleagues the large majority of the participants (90%) that are adopting avoidance strategies to prevent further relapses, stated that they based their choices mostly on their own experience with certain products (Crooks et al., 2021), even though there are also several accounts of patients that received dietary advice from healthcare professional; the most consulted were the dietician and the gastroenterologist (Limdi et al., 2016; Marsh et al., 2019; De Vries et al., 2019; Zallot et al., 2013). In contrast to these studies, De Vries and colleagues identified internet being the most consulted source of information regarding food for IBD patients, followed by a dietician and the treating medical specialist in the hospital (De Vries et al., 2019). In Marsh et al., participants with active disease showed greater confidence in advice obtained from internet, followed by the gastroenterologist, the dietician and the general practitioner; patients currently in remission, on the other hand, were more confident with the advice received from the gastroenterologist, compared to both the dietician and the internet (Marsh et al., 2019). In their study, Limdi and colleagues showed that half of their participants never received any nutritional advice (Limdi et al., 2016). This trend seems different for paediatric patients: one study indeed reported that almost all parents of children with IBD (91%) had received nutritional instructions and were more likely to rely on advice given by doctors (74%) and dieticians (70%), but many of them also admitted to search information from nonprofessional sources (85%) (Pituch‐Zdanowska et al., 2019). Guadagnoli and colleagues reported that patients likely use self‐directed dietary therapies and that less than half (41%) of IBD participants had ever met a dietician for a prescribed diet; meeting with a dietician, however, not always improved the food‐related quality of life of patients (Guadagnoli et al., 2019).

While this difficulty in accessing information regarding diet and food is not reported in all the included studies (actually, some studies reported that most participants found dietary advice easily available and were almost satisfied about it Pituch‐Zdanowska et al., 2019; De Vries et al., 2019), in other studies many patients expressed concerns regarding their limited knowledge about how diet affects their IBD: in one study, for instance, the patients found advice received from their gastroenterologists and dieticians often vague and not very useful, and sometimes even conflicting (Czuber‐Dochan et al., 2020); while the majority of participants in a second study reported that they had to find out for themselves which dietary habits suited them the best, and that they received this very same advice from their own healthcare providers: not having access to a reliable source of information, and having instead to rely on try‐and‐error strategies, was however reported as a source of uncertainty and frustration (Palant et al., 2015).

Finally, the restrictions to the diet also impact on the ‘pleasure of eating’ that IBD patients experience. Only three quantitative studies investigated how IBD influenced the appetite and the pleasure of eating, but in these studies about half the adult participants (45%–66%) stated that the disease affected their appetite and their pleasure of eating and that they forced to deprive themselves of foods they really like in order to prevent relapse (Limdi et al., 2016; Marsh et al., 2019; Zallot et al., 2013). Authors found that appetite decreased during relapse, compared to remission; furthermore, the loss of pleasure and appetite was more evident in CD patients (87%), compared to UC patients (66%) (Limdi et al., 2016). Parents also reported negative effects of the disease on the enjoyment of food of their children with IBD (Pituch‐Zdanowska et al., 2019). Food avoidance is perceived as a sacrifice also by paediatric patients. One study reported that one parent out of three believes that their children are avoiding some dietary products they really like because of the fear of exacerbation of symptoms, and parents of children with a shorter history of IBD tended more often to believe that their child derives less pleasure from food than before the onset of the disease. The products identified by parents as more ‘painful to avoid’, precisely because are usually the ones preferred by young patients, are fried dishes, sweets, fast food, milk and dairy products, and salty snacks.

#### 3.3.2 Social life

The need to avoid certain foods and to pay attention to food intake not only affects people's personal life and their psychological wellbeing but also has an impact on their social lives. For instance, one study reported that 44% of the interviewed parents of IBD patients consider the disease and the risk of flares as the main reason why their children avoid eating out; in particular, girls tend to refuse outdoor dining more often than boys (Pituch‐Zdanowska et al., 2019). This might be exacerbated in those ethnic communities where sharing food has a stronger cultural significance: many interviewed patients reported having to renounce to participate in ceremonies and social events where food had a cultural relevance (Alexakis et al., 2015).

This is not only true for young patients, but also for adults, as different studies highlighted how the work environment and social events can create important challenges for people with IBD (Alexakis et al., 2015; Czuber‐Dochan et al., 2020; Palant et al., 2015): for instance, some patients felt they had to control their disease by preparing their own lunch at home, by eating less or by directly avoiding eating when with their colleagues (Czuber‐Dochan et al., 2020). The fact that patients need to eat at very well‐regulated times with well‐regulated portion sizes also affects their possibility to enjoy a meal out of home (De Vries et al., 2019). It has been reported that almost one patient out of four (21%–23%) refuses dining out of home in order to prevent relapses (Crooks et al., 2021; Limdi et al., 2016; Zallot et al., 2013).

#### 3.3.3 Family sphere

Finally, the need for a specific diet also impacts patients’ family lives. Regarding young patients, it has been reported that some parents, or whole families, adapted to their child's diets, thus renouncing the same foods that are potentially harmful to the patients themselves, or they might prepare separate dishes, but often these are only slightly different, in this way the patient would have the perception to eat ‘the same thing’ as the other members of the family (Chuong et al., 2019). The dietary practices and restrictions of the young patients seem to be considered very important by parents of paediatric patients: in a qualitative study (Chuong et al., 2019), some of the parents of children with IBD stated that they tried to maintain a ‘normal’ routine of food practices for their family, while also supporting their children's needs due to their IBD: indeed, many parents interviewed reported being careful during grocery and buying mainly products they considered healthy.

On the other hand, in other studies, between 19% and 28% of adult patients declared that they do not generally share the same menu as the other members of the family, and have a tendency to cook and eat different meals, even though this was sometimes perceived as stressful (Czuber‐Dochan et al., 2020; Palant et al., 2015; De Vries et al., 2019). In one qualitative account, this was reported to be actually coupled with the sense of guilt from the young participants, as they reported feeling like a burden for their own families due to the special effort required to accommodate their needs (Alexakis et al., 2015).

## 4 DISCUSSION

The role of nutrition in determining chronic diseases evolution is more and more acknowledged by the scientific community. In the case of gastrointestinal diseases such as IBD, this link seems even more evident. This makes particularly relevant the role of patients’ food choices as a crucial factor that impacts the clinical evolution of the disease. In particular, the role of patients’ food choices is more and more regarded as important in the management of IBD, both because impacting on the disease evolution and on the symptoms’ management (Green et al., 2019; Hou et al., 2014; Kinsey & Burden, 2016; Mehrabani et al., 2017). Not only the scientific community is increasingly recognising the role of food choices in IBD management, but also patients’ experiences more and more confirm the perceived impact of their nutritional choices on their symptoms (Cohen et al., 2013; Limdi et al., 2016; Marsh et al., 2019; De Vries et al., 2019). Although important, the nutritional guidelines to which IBD patients should adhere in managing their diet are still rather unclear and debated. The consequence is that patients may follow ‘self‐directed’ diets, choosing food according to their emotional and irrational beliefs, rather than by a clinical understanding of the nutritional impact on their disease.

In the articles included in our review, we found that ‐although there is no clear consensus‐ the foods generally avoided by IBD patients are spicy and fatty foods, carbonated drinks, and milk with its derivates. Other foods that are often reported as avoided are raw vegetables and fruits, along with foods with a high fibres content. This is in line with the results from a previous systematic review on a dietary recommendation for IBD patients: the authors found that spicy and fatty food, raw vegetables, dairy products, and food with a high content in fibres overall are oftentimes discouraged (Hou et al., 2014).

An important driver of such patients’ food choices (and related spontaneously oriented food restrictions) is the patients’ perceived impact of diet on the quality of life: the consequences of food consumption in terms of perceived psychological wellbeing, social inclusion and quality of life are often at the basis of the patients’ personal decisions in terms of their diet. Nevertheless, there is not much research on the psychosocial impact that food restrictions and strategies exert on patients: current studies are mainly based on a qualitative research design and there is a lack of more quantitative scientific evidence on this matter. Our scoping review aimed at systematising the scientific debate about food‐related choices impact on IBD patients’ perceived quality of life, in order to cast light on the elements which need further exploration and intervention in order to orient healthier and safer nutrition.

The literature retrieved confirmed how patients share the belief that food is an important ‘ingredient’ in determining the outcomes of their disease (Cohen et al., 2013; Limdi et al., 2016; Marsh et al., 2019; De Vries et al., 2019), thus engaging in food restrictions and avoidance for ingredients which their subjectively believe as negative (Crooks et al., 2021; Limdi et al., 2016; Marsh et al., 2019; De Vries et al., 2019; Zallot et al., 2013). Both the worry for the potential consequences of food consumption on symptoms severity and the self‐imposed restrictions, often lead patients (particularly the younger ones) in experiencing isolation, discrimination and social exclusion (Alexakis et al., 2015; Pituch‐Zdanowska et al., 2019). Even with relatives and within the family, young patients report the negative impact of self‐imposed food restrictions on the quality of their relationships: indeed, there are accounts of complex familiar situations such as the need to invest time and effort in preparing separated and the consequent feeling of ‘exclusion’ from normal family dynamics and of sense diversity (Czuber‐Dochan et al., 2020; Palant et al., 2015; De Vries et al., 2019).

Literature shows how patients tend to develop personal strategies to manage their diet and to avoid foods perceived as ‘dangerous’. However, this comes to a cost since patients have to be very careful about what, when, and how much they eat. This, again, leads to more difficult management of social relationships and personal time (Czuber‐Dochan et al., 2020).

Another source of distress (indirectly) related to food for IBD patients is the source of information: several studies report that patients, in order to develop their ‘knowledge’ and expertise of the foods they need to avoid, have to mostly rely on personal experience, trial and error, and sometimes even of potentially untrustful sources of information such as the web (Guadagnoli et al., 2019; Limdi et al., 2016; Pituch‐Zdanowska et al., 2019; De Vries et al., 2019).

The anticipatory fear of incurring in symptoms related to food consumption, the sense of losing control over one's own nutrition and quality of life together with the experience of social isolation tend to have a negative impact on the psychological wellbeing of patients: many patients feel frustrated and annoyed by having to plan ahead due to their peculiar food routines, which might cause non‐adherence and a worsening of symptoms (Czuber‐Dochan et al., 2020; Palant et al., 2015).

Furthermore, the studies retrieved show that patients strive for a more systematic approach to diet management in IBD care: both in terms of receiving clearer information and guidelines for managing one's own nutrition and in terms of pragmatic support in order to become better success in their food consumption decisions (Chuong et al., 2019; Czuber‐Dochan et al., 2020; Limdi et al., 2016; Palant et al., 2015; De Vries et al., 2019).

Finally, it is important to notice that almost all of the studies included in our review are from either the UK, other European countries (Netherlands, France, Germany, Poland), or countries of ‘western’ culture (USA, Canada, Australia). Moreover, no studies compared populations from different cultures. Only one study (Alexakis et al., 2015), among the ones included, focuses on the peculiarities of IBD patients of UK cultural minorities (i.e. black or south Asian background): results indeed show the importance of the cultural background in determining the relationship with food of the participants.

This is consistent with the existing literature on food choices, and particularly relevant, as several studies show the importance of culture on food choices, attitudes, and habits in the general population (see, for example, Risso et al., 2017; Rodríguez‐Arauz et al., 2016; Rozin, 2005). Future studies show be aware of the relevance of the cultural background and try to fill the current gap in understanding how cultural differences might influence the way food restrictions impact IBD patients.

This scoping review was the first attempt into the literature of synthesising the increasing scientific debate about the role of food consumption in impacting IBD patients’ quality of life. This analysis cast light on the current gaps in the literature: in particular, it appears evident the need for more studies addressing the psychosocial impact of diet on IBD patients’ sense of wellbeing and how this may be at the basis of their nutritional conduct. The analysis of false beliefs, emotional distress and social isolation on food choices and how this effectively impacts on symptoms control would be further investigated in a better systematic way. Furthermore, the results of this study highlight the importance of orienting multidisciplinary research endeavours in this area, by putting into dialogue psychological theories, with the nutritional and clinical ones. Finally, there is the need for studies focused on younger patients, for whom the social impact might be more relevant from a developmental point of view.

### 4.1 Limits

This study has some limitations. First and foremost, only articles written in English and retrievable by the authors were included in the screening process which might have led to the exclusion of some potentially relevant studies. Furthermore, only a selection of scientific databases was included in the search strategy, potentially limiting the number of articles retrieved. Finally, this is a review with a mostly descriptive purpose, as given the nature of the data it was not possible to do a meta‐analysis. This clearly limits the extent of the conclusions that can be drawn, and the data that were collected could be biased due to their nature (as they are retrieved mostly from patients’ perspectives and interviews). Moreover, given the nature of the scoping reviews, there are some limitations to this methodology: this study only summarises and describes significative results from quantitative (and qualitative) studies, thus reporting a potentially biased account of evidence. Readers should be aware of this possible bias in evidence selection. Nevertheless, scoping reviews allow a flexible summarisation of the ‘state of art’ of a field, have the potential to highlight gaps in the scientific literature regarding a certain topic, and are particularly appropriate in addressing broad research questions like the one presented in this paper (Arksey & O’Malley, 2005).

## CONFLICT OF INTEREST

The authors declare that there is no conflict of interest to disclose.

## AUTHOR CONTRIBUTIONS

LP and PF equally contributed to data collection and analyses, as well as the original drafting of the manuscript. LP and GG designed the study methodology. GG and SL were in charge of supervision, project and funding management. All authors critically revised the manuscript and accepted its final form.

## Data Availability

No new data were generated or analysed in support of this research.
